# The Biosafety Research Road Map: The Search for Evidence to Support Practices in the Laboratory—Foot and Mouth Disease Virus

**DOI:** 10.1089/apb.2022.0041

**Published:** 2023-12-05

**Authors:** Stuart D. Blacksell, Sandhya Dhawan, Marina Kusumoto, Kim Khanh Le, Kathrin Summermatter, Joseph O'Keefe, Joseph Kozlovac, Salama Suhail Almuhairi, Indrawati Sendow, Christina M. Scheel, Anthony Ahumibe, Zibusiso M. Masuku, Allan M. Bennett, Kazunobu Kojima, David R. Harper, Keith Hamilton

**Affiliations:** ^1^ Mahidol-Oxford Tropical Research Medicine Unit, Faculty of Tropical Medicine, Mahidol University, Bangkok, Thailand.; ^2^ Centre for Tropical Medicine and Global Health, Nuffield Department of Medicine, University of Oxford, Oxford, United Kingdom.; ^3^ Institute for Infectious Diseases, University of Bern, Bern, Switzerland.; ^4^ Ministry for Primary Industries, Wellington, New Zealand.; ^5^ United States Department of Agriculture, Agricultural Research Service, Beltsville, Maryland, USA.; ^6^ Abu Dhabi Agriculture and Food Safety Authority, Abu Dhabi, United Arab Emirates.; ^7^ Indonesian Research Center for Veterinary Science, Bogor, Indonesia.; ^8^ WHO Collaborating Center for Biosafety and Biosecurity, Office of the Associate Director for Laboratory Science, Center for Global Health, Centers for Disease Control and Prevention, Atlanta, Georgia, USA.; ^9^ Nigeria Centre for Disease Control and Prevention, Abuja, Nigeria.; ^10^ National Institute for Communicable Diseases of the National Health Laboratory Services, Johannesburg, South Africa.; ^11^ UK Health Security Agency, Porton Down, United Kingdom.; ^12^ Department of Epidemic and Pandemic Preparedness and Prevention World Health Organization (WHO), Geneva, Switzerland.; ^13^ The Royal Institute of International Affairs, London, United Kingdom.; ^14^ World Organisation for Animal Health (OIE), Paris, France.

**Keywords:** foot and mouth disease, pathogen characteristics, biorisk management, biosafety evidence, biosafety knowledge gap analysis

## Abstract

**Introduction:**

Foot and mouth disease (FMD) is a highly contagious infection of cloven-hoofed animals. The Biosafety Research Road Map reviewed scientific literature regarding the foot and mouth disease virus (FMDV). This project aims to identify gaps in the data required to conduct evidence-based biorisk assessments, as described by Blacksell et al., and strengthen control measures appropriate for local and national laboratories.

**Methods:**

A literature search was conducted to identify potential gaps in biosafety and focused on five main sections: the route of inoculation/modes of transmission, infectious dose, laboratory-acquired infections, containment releases, and disinfection and decontamination strategies.

**Results:**

The available data regarding biosafety knowledge gaps and existing evidence have been collated. Some gaps include the need for more scientific data that identify the specific safety contribution of engineering controls, support requirements for showering out after in vitro laboratory work, and whether a 3- to 5-day quarantine period should be applied to individuals conducting in vitro versus in vivo work. Addressing these gaps will contribute to the remediation and improvement of biosafety and biosecurity systems when working with FMDV.

## Introduction

The World Organisation for Animal Health (WOAH, formerly OIE), the World Health Organization (WHO), and Chatham House are currently collaborating to improve the sustainable implementation of laboratory biological risk management, particularly in low-resource settings under the banner of the Biosafety Research Road Map (BRM) project. The BRM project aims to improve laboratory sustainability by providing an evidence base for biosafety measures (including engineering controls) and evidence-based biosafety options for low-resource settings. This will inform strategic decisions on global health security and investments in laboratory systems. This work involves assessing the current evidence base required for implementing laboratory biological risk management, aiming to provide better access to evidence, identifying research and capability gaps that need to be addressed, and providing recommendations on how an evidence-based biorisk management approach can support biosafety and biosecurity in low-resource settings.

This article presents the characteristics of the foot and mouth disease virus (FMDV), the current biosafety, biosecurity, and biocontainment evidence, and the available information regarding laboratory-acquired infections and laboratory releases.

## Materials and Methods

A 15-member technical working group (TWG) was formed to develop a BRM to support the application of laboratory biological risk management and improve laboratory sustainability by providing an evidence base for biosafety measures. The TWG conducted a gap analysis for a selected list of priority pathogens on procedures related to diagnostic testing and associated research for those pathogens, including but not limited to sample processing, testing, animal models, tissue processing, necropsy, culture, storage, waste disposal, and decontamination. The TWG screened databases, websites, publications, reviews, articles, and reference libraries for relevant data to achieve this. The main research domains used to perform the literature searches were the ABSA database, Belgian Biosafety Server, CDC reports, WHO reports, PubMed, and internet searches for terms related to biosafety matters, including, for example, inactivation, decontamination, laboratory-acquired infections, laboratory releases, and modes of transmission.

The summary of evidence and potential gaps in biosafety was divided into five main sections: route of inoculation/modes of transmission, infectious dose, laboratory-acquired infections, containment releases, and disinfection and decontamination strategies. Blacksell et al.^1^ described the materials and methods and explains why the gap analysis was performed.

## General Characteristics

Foot and mouth disease (FMD) is a highly infectious viral disease caused by the FMDV belonging to the Picornaviridae family, a positive-sense, single-stranded RNA virus. FMDV primarily infects cloven-hoofed animals and is transmissible by aerosols and droplets,^2–4^ and indirect (fomites) or direct contact.^5^

There are seven immunologically distinct FMDV serotypes (O, A, Asia-1, C, SAT-1, SAT-2, and SAT-3), of which six of the seven serotypes (O, A, C, SAT-1, SAT-2, and SAT-3) have occurred in Africa. In comparison, Asia has four serotypes (O, A, Asia-1, and C, with the three former serotypes dominating), and South America with only three (O, A, and C).^6,7^ The Progressive Control Pathway for FMD,^8^ one of the core tools of the Global FMD Control Strategy, emphasizes the importance of implementing effective biosecurity practices, hygiene, cleaning, and disinfection routines. Most FMD control guidelines focus on facilities in countries where FMD is exotic, which results in highly stringent containment requirements with the primary objective^2–4^ of preventing the release of the virus into the environment. In non-endemic settings, FMDV is classified as a risk group 4 in the European Union (EU),^9,10^ a 3Ag in the United States [*Biosafety in Microbiological and Biomedical Laboratories* (*BMBL*), sixth edition^11^], and a Select Agent pathogen.^12^

In FMD containment infrastructures, the following activities have to be considered:

- In vitro laboratory activities (diagnostic, FMD contingency laboratories, research) with primary containment devices (e.g., biological safety cabinet) and dedicated personal protective equipment (PPE), usually small virus quantities
- In vivo activities (e.g., housed large animals) where the room is considered the primary barrier
- Large-scale facilities (e.g., vaccine production) that mainly use closed vessels with large amounts of infectious FMDV

### Local/National FMD Situation

Biosafety and biosecurity standards should be proportionate to the disease situation in the country or zone of the facility location. The EU-FMD Minimum Biorisk Management Standards for laboratories working with FMDV^10^ distinguish between four tiers of biorisk, depending on the local/national FMD situation. Guidelines for Tier D and Tier C laboratories have been published.

- Tier A: General diagnostic laboratories in FMD-endemic countries
- Tier B: Laboratories working with infectious FMDV in FMD-endemic countries
- Tier C: Laboratories undertaking diagnostic investigations for FMDV in the framework of a national contingency plan in FMD-free countries
- Tier D: (Inter)national FMDV reference laboratories working with infectious FMDV in FMD-free countries

The spread of FMD in a region or country and the economic situation are important factors that must be considered in the risk assessment.

### Treatment and Prophylaxis

Prophylaxis and control of FMD are achieved via vaccination in endemic countries.^3,13^ The vaccine must be effective against the viral serotype and subgroup causing the outbreak, as there are 7 known types and more than 60 subtypes of FMDV. Immunity to one type does not protect an animal against other types or subtypes.^14^ In countries where FMD is exotic, slaughter and ring vaccination may be used to stamp out the disease. However, this will be dependent on the regulations of the individual jurisdiction.

### Diagnostics

Laboratory procedures for diagnosing FMD include indirect double-antibody sandwich antigen detection enzyme-linked immunosorbent assay (ELISA), virus isolation/identification, and real-time polymerase chain reaction (RT-PCR).^15^ Until recently, antigen detection ELISA was widely used as it was relatively simple to perform, reasonably rapid (∼2–3 h), and could provide a diagnosis to the serotype level. However, specificity and reagent supply problems have increased the application of RT-PCR. Virus isolation is not routinely performed for diagnostic purposes, however, may be required for vaccine selection/matching purposes. All tissue grinding at the initial stages of the diagnostic process is performed in a class II biological safety cabinet to minimize the spread of aerosols.

## Biosafety Evidence

### Modes of Transmission

In animal-to-animal transmission, the most common mechanism of spread of FMDV is by direct contact initiated by the deposition of droplets or droplet nuclei (aerosols) in the respiratory tract or by the mechanical transfer of virus from infected to susceptible animals and subsequent virus entry through cuts or abrasions in the skin or mucosae. Transmission of the virus may also occur indirectly via any contaminated surface or product (adapted from Alexandersen and Mowat^16^). The virus is considered highly pathogenic as it can survive in the environment without animal hosts.^17,18^ Potential virus reservoirs include the excretions and secretions of infected livestock and contaminated inanimate objects or fomites.^2,19,20^ Humans acting as vectors can also be vital in spreading FMDV.^21–28^

Under specific epidemiological, climatic, and meteorological conditions, short-distance aerosol transmission, which, as mentioned above, is a highly efficient route of infection of ruminants, may be extended to airborne transmission over a significant distance. This is mainly a risk when large numbers of pigs are infected because pigs excrete large quantities of airborne virus (up to 10^5.6^–10^8.6^ 50% tissue culture infectious doses [TCID_50_] per pig per day). Ruminants excrete less virus in their breath (10^4^–10^5^ TCID_50_ per day)^16^ but, in contrast to pigs, are highly susceptible to infection by inhaled virus.^4,29,30^

Humans can transmit FMDV to susceptible animals via fomites (e.g., via contaminated clothing, footwear). Typically, in many facilities, no or only minimal PPE is used when handling large animals infected with FMDV. The room is considered primary containment in these animal units, and personnel working inside these rooms are exposed to FMDV. There is evidence that hygiene measures such as changing clothes and shoes, handwashing, or showering prevent the transmission of FMDV to other animals.^21,26–28,33^ It has also been shown that the virus can survive up to 24–48 h in the nose of persons handling infected animals.^21,26,27,33^

### Infectious Dose

It has been established that ruminants can be infected experimentally by airborne exposure to only 10 TCID_50_, whereas to infect pigs by this route, more than 10^3^ TCID_50_ are required, and infection only occurs if the virus is delivered at a high concentration^29–32^ (adapted from Alexandersen and Mowat^16^).

### Human Susceptibility and Laboratory-Acquired Infections

Human susceptibility to FMDV has been debated for many years; however, the virus has been isolated and typed (type O, followed by type C and rarely A) in more than 40 human cases.^34^ FMD infection in humans appears rare, and predisposing factors, including proximity, wounds, and a high exposure intensity, play a crucial part in initiating clinical signs.^35^ In the review of human FMDV infections by Hyslop,^35^ numerous cases were reported, with 1 report from 1834 of 3 veterinarians acquiring FMD after deliberately drinking raw milk from infected cows and another 22 cases following the consumption of infected milk.

Another human FMD case was reported following a hand wound from a broken vial containing FMDV during an animal experiment at a research facility in Germany in 1921 (Table 1).^36^ Occupational FMD was also reported from a butcher's table in Poland in 1938 (Table 1).^24^ In the 1966^37^ and 2011^23^ UK-FMD outbreaks, humans near FMD animal cases developed FMD-like symptoms; however, the infections were not laboratory confirmed. Human cases after exposure to sick animals have been reported, although many are historical reports with no information about comorbidities.^22,24,38^

**Table 1.** Detailed pathogen biosafety evidence for foot and mouth disease virus

Method	Details		Evidence (direct quote where available)	Ref.	Evidence gap? (yes/no)
Modes of transmission	Human to animals	Human respiratory tract—positive transmission	“Entered in another stable, put-on new clothes, sneezed and coughed at nostrils of animals (30 sec).” “Man can be a hazard in the spread of FMD, virus disappears 48 h after exposure.”	^27^	No—conditions represent experimental infections, not natural infections
			“The infection was assumed to have passed via the nasal cavity. However, this required prolonged contact with infected pigs and deliberate coughing, blowing and sneezing on the muzzles of the susceptible cattle” (Auty et al.^17^ referring to the Sellers et al.^27^ experiment)	^17^	
			“To my knowledge only one instance of human nasal transmission of FMD virus from infected to non-infected animals has been recorded (Sellers and others 1971), and to put this finding in the context of probability and dose it is necessary to give some details. The transfer took place under the following experimental conditions. Four researchers performed clinical examinations on groups of eight pigs with clinical FMD in an isolation compound. On completion of the examination the rubber kits worn by each of the examiners, together with their hands, were sprayed with a 4 per cent sodium carbonate liquid soap and water solution. After removal of the rubber kits the examiners washed their faces and hands with soap and water and scrubbed their nails. They took off their laboratory clothes, had a shower and put on their outside clothes. The examiners then walked to another separate isolation unit, where they took off their out-side clothes, put on clean clothes and a rubber kit. Susceptible, non-infected cattle were housed two to a loose box in the unit, and the examiners entered the loose box and examined the animals, at the same time sneezing, snorting, coughing and breathing at the muzzles of the animals. The exposure of each animal to this treatment lasted 30 seconds for each person. The interval between the examination of the pigs and cattle ranged from 15 to 22 minutes. The experiment was repeated. In the first experiment, the upper half of the door of the loose box was left open and the air flow of the filtration system maintained. In the second experiment, the inlet and outlet of the air to the loose box were blocked and the walls were sprayed with water. no lesions when it was euthanased on the 16th day. It was concluded that this animal had been infected by the animal that showed lesions on day 14. These findings, in particular the 14-day incubation period, show that although human nasal transmission occurred in one of four cattle exposed, the dose transmitted was extremely low, despite the exposure of examiners to pigs at a time when airborne virus excretion was at its maximum level) and the strenuous efforts by examiners to release virus from their respiratory tracts.” (Donaldson referring to the Sellers et al.^27^ experiment).	^51^	
		Human respiratory tract—negative transmission	“Sampling of human subjects, who had been in contact with animals infected with foot-and-mouth disease (FMD) virus, showed that virus could be recovered from the nose, throat, saliva and from air expelled during coughing, sneezing, talking and breathing. The amounts of virus recovered paralleled those collected with a large-volume sampler and multistage impinger and these findings confirmed that the highest recovery of airborne virus was from infected pigs followed by cattle and sheep. More virus was found in the noses of those examining infected animals than in those operating the samplers, but there was variation between the subjects. In the majority there was a 1.8 log fall in titre by 3.5 hr., but virus persisted in the nose of one subject for 28 hr.” “Nose blowing or washing the nostrils did not remove virus completely, nor were cloth or industrial masks completely effective in preventing inhalation of virus. It was possible to transmit virus from infected subjects to others on one occasion. No clinical cases of FMD in man resulted from exposure, nor was there any rise in antibody.”	^26^	No
			“The present results indicate a low risk of virus survival in the nasal cavities of personnel 16 to 22 hours after exposure to infected animals. Variation in the extent of contact with infected animals in the field might influence nasal contamination of personnel and virus survival within the nasal cavities. However, the close contact with infected animals and enclosed sampling environment of the four experiments, especially when the room ventilation was shut down, might be expected to have resulted in higher concentrations of airborne virus, compared with the virus concentration level found in the field.”	^28^	
			FMDV was not detected in nasal secretions of investigators.	^21^	
		Showering and clean outerwear	“Rubber kits and hands sprayed with 4% sodium carbonate-liquid soap, faces and hands washed, nails scrubbed with soap, removal of clothes and showering.”	^27^	Yes—no evidence to support showering by staff following *in vitro* FMDV laboratory work. Data presented only following work with FMDV-infected animals.
			“To test the effectiveness of biosecurity procedures in preventing the transmission of FMD virus (O/UK/35/2001) investigators contacted and sampled pigs inoculated with FMD virus for approximately 45 minutes and then contacted and sampled sentinel pigs and sheep after either using no biosecurity procedures, or washing their hands and donning clean outerwear, or showering and donning clean outerwear. The virus was detected in the nasal secretions of one investigator immediately after the post-mortem investigation of the inoculated pigs but was not detected in samples collected between approximately 12 and 48 hours later. After the contaminated personnel had showered and changed into clean outerwear, they did not transmit the strain of FMD virus to susceptible pigs and sheep.” “Only handwash and clean outerwear was sufficient to prevent mechanical transmission.”	^33^	
			“Investigators contacted and sampled FMDV-inoculated pigs for approximately 40 min and then contacted and sampled sentinel pigs after using no biosecurity procedures, washing hands and donning clean outerwear, or showering and donning clean outerwear. Personnel were sampled for nasal carriage of FMDV for 85.43 h. Contaminated personnel did not transmit FMDV to susceptible pigs after handwashing or showering, and donning clean outerwear. FMDV was transmitted when biosecurity procedures were not used.”	^21^	
		Quarantine	“Thus, extended animal avoidance periods do not appear to be necessary to prevent transmission of FMDV (O/TAW/97) by people to pigs when organic material is removed through handwashing/showering and donning clean outerwear. This study supports similar findings in a previous publication using FMDV (O/UK/35/2001).”	^21^	Yes—there is no evidence that the length of time inside a room affects transmission.
		Masks	“FMD virus excretion in breath occurs before the appearance of lesions in affected species and can peak in bovine breath before lesions appear. Veterinary staff closely examining the tongues of such lesion-free cattle on what appear to be uninfected premises could inhale moist bovine breath containing virus.” “Veterinarians involved in surveillance can routinely visit eight different premises a day during an extensive epizootic.”	^51,53,54^	Yes—effect of respiratory personal protective equipment on virus uptake by humans after handling FMDV-infected animals, the evidence is inconclusive. In order to prevent accidental transmission, additional research is required on the effects of nose blowing and nasal washing.
	Animal to animal	Direct and indirect contact	“Direct contact experiments, contact calves were exposed to inoculated calves in the same room. In indirect contact experiments, contact calves were housed in rooms that previously had held inoculated calves for three days (either from 0 to 3 or from 3 to 6 days post inoculation). Secretions and excretions from all calves were tested for the presence of FMDV by virus isolation; the results were used to quantify FMDV transmission.” “Results show that roughly 44% of the transmission of FMDV occurs via the environment, in the days after the calves started secreting and excreting the virus. The contribution of the environment to the transmission of FMDV depends on the FMDV survival rate; if the survival rate is high, the contribution of the environment is higher.”	^56^	No
			“The majority of average survival estimates listed in Tables 1 and 2 are three months or less. This tentatively supports the three-month rule for regions with hot (>20°C) or, possibly, temperate (4 to 20°C) climates. The initial virus titre of the infected or contaminated material, the susceptibility of the livestock exposed to virus and their degree of exposure must also be considered when assessing the risk posed by that material.”	^57^	
			“Our present and previous studies indicate that (a) horizontal transmission between animals of the same species occurs more easily than that between animals of different species”	^58^	
			“In-pen contact pigs and sheep (groups 2 and 3) All five of the in-pen contact pigs developed gross lesions consistent with FMD, and FMD virus was detected in all the samples of blood and the nasal swabs collected when they were euthanized two days after their exposure.”	^33^	
	Aerosol		“Aerosol inoculation of FMDV consistently resulted in virus detection by real-time reverse transcriptase-polymerase chain reaction and viral isolation in the soft palate, pharynx, and lungs. Viral antigen was also detected in each of these tissues by immunohistochemistry. Aerosol exposure resulted in typical clinical signs of FMD when animals were kept alive long enough to develop disease.”	^59^	No
			“The strains of FMD virus examined in this study, in the form of aerosols from milk and faecal slurry, were much more stable than was found in previous investigations.”	^2^	
			“These experimental results, namely the high output of airborne virus by pigs and the extreme sensitivity of cattle to respiratory infection, provide an explanation for the findings of field studies where the pattern of spread of FMD over long distances has invariably been from infected pigs at source to cattle downwind.”	^60^	
			“The air in looseboxes containing groups of pigs in the acute stage of foot-and-mouth disease was sampled simultaneously with two air-sampling devices: a large volume sampler (Litton) and a cyclone sampler. Although the cyclone sampler was slightly less efficient at trapping airborne virus it was easier to operate. When pigs were sampled individually within a 610 litre cabinet using the cyclone sampler, the mean recovery of virus over a 24 hour period was log10 8 × 6 TCID_50_ per animal. This figure is four times higher than the maximum amount estimated in previous studies in which groups of pigs held in looseboxes were sampled with a Litton apparatus.”	^61^	
			“The highly contagious nature of FMD is a reflection of the wide range of species which are susceptible, the enormous quantities of virus liberated by infected animals, the range of excretions and secretions which can be infectious, the stability of the virus in the environment, the multiplicity of routes of infection and the very small doses of virus which can initiate infection in susceptible hosts”	^3^	
	Droplets		“Airborne virus is excreted mainly in the exhaled breath of infected animals as droplets and droplet nuclei originating from the upper and later from the lower respiratory tract”	^29^	No
	Fomites		“Virus can survive in slurry for up to 9 days at 20°C, to 14 weeks at 5°C”	^17,18^	No
Infectious dose—animals	Pigs	OPF 10^2.1–103.1^ TCID_50_/mL Lowest dose to cause lesions: 125 IU	“For contact exposed animals, the Category I pigs, which had been exposed to donors from 8 to 24 hpi, were not infected despite consistent detection of shedding of low quantities of FMDV from the donors. The estimated range of FMDV infectious dose shedding in OPF over this time period was 10^2.1–103.1^ TCID_50_/ml.”	^62^	No
			“The lowest dose to cause infection in pigs exposed to natural virus through a mask was log 1.35 IU (22 IU), while the lowest dose to cause lesions was log 2.1 IU (125 IU)”	^63,64^	
	Sheep	Lowest dose for instillation: log 3.35 IU (2250 IU)	“Strains of types O, A and C were used in the experiments the majority being of type O. End points could not be demonstrated in every experiment, some showing successful infection, one other failure to infect by indirect contact (J.-F. Valarcher et al., unpublished data). The lowest dose for instillation was log 3.35 IU (2250 IU)”	^63^	
	Cattle	Lowest dose for instillation: 100 IU Lowest dose by spray: 90 IU	“The lowest dose by instillation was found to be log 2.0 IU (100 IU) (Sutmoller et al., 1968) and by spray was log 1.95 IU (90 IU)”	^63^	
LAIs and human infections	Miscellaneous human FMD cases 1921–1969		“Beginning in 1921 up to 1969 at least 38 papers were published, which described clinically manifest FMD in man in more than 40 proven cases.” “Proven cases of FMD in man have occurred in several countries in Europe, Africa, South America. The type of virus most frequently isolated man is type O followed by type C rarely A.”	^34^	No
	Germany 1834		“There is one report from 1834 of three veterinarians acquiring the disease from deliberately drinking raw milk from infected cows”	^37^	
	United Kingdom, 1966		“The last human case reported in Britain occurred in 1966, during the last epidemic of foot and mouth disease.^4^ The circumstances in which it does occur in humans are not well defined, though all reported cases have had close contact with infected animals.”	^37^	
	Laboratory accident at Insel Riems research institute, Germany 1921. Description by Dr. J. Pape who experienced the incident		(Translation from German) As the result of carelessness, the hind legs were one poorly bound pig, had so that it has so much movement that it had freedom to crush the glass vial I was holding causing it to shatter. A shard of glass entered my right hand and caused an approximately 2.5 cm long cut, very deep wound that bled profusely. After about 5 minutes long flushing in Lysol solution was the wound was sewn. The healing process was normal. Two days after the injury I felt headache and slight malaise for a short time persistent chills. On the morning of the third day, I felt walking a pain in the heel of the right foot and there on examination found a roughly bean-size large blister attributed to boot rubbing. Around noon began to develop blisters on hands and feet. The vesicle formation stopped after 2 days. It arose at the plantar surfaces of the hands and feet, altogether about 25 vesicles from the size of a lentil up to that of a cherry stone. I opened the vesicle and a water-clear, faintly amber stream poured out similar to the yellow liquid, such as we find in the vesicle of sick cattle, pigs and guinea pigs, but not pus, which some authors in the Foot and Mouth Epidemic claim to have seen from aphthous ulcers. After lifting, the cherry-red bottom of the vesicle became visible, which is still secreted profusely for several hours. The epithelization of the erosion took place rapidly, so that in 4–5 days the healing was complete. Simultaneously with the formation of vesicle on hands and feet there was a slight, slightly painful gum irritation that disappeared after 2 days. There was no formation of vesicle or erosion. Tongue and lips remained altogether free from any inflammatory eruption appearance.	^36^	
			“Nevertheless, one author developed symptoms of FMD after cutting himself on a broken vial containing FMD virus”	^35^	
	University Clinic of Lwow. Poland, 1938. Described in Polska Gazeta Lekarska (1938, 17, 501)		“A man aged 40 was wounded in the finger by a splinter from a butcher's table. The wound, although slight, healed very slowly. A transitory lymphangitis and lymphadenitis followed in a few days in the affected arm. Two weeks later, general weakness, headache, and febrile manifestations developed, the temperature rising in a few hours to 102′F. These symptoms persisted for three days, the patient believing himself to be suffering from ‘influenza.’ On the third day, painful mastication and salivation ensued; multiple vesicles appeared in the mouth, later giving way to ulcer formation. The following day the ulcerations were found to be larger and were bleeding. The doctor, reconsidering his original diagnosis of ‘aphthous stomatitis,’ suspected an acute leukaemia oragranulocytosis.” “Guinea-pigs inoculated with the serous fluid from the soles and palms developed the typical signs and symptoms of foot and mouth disease after 12 to 14 days.”	^24^	
	FMD outbreak UK, 2001		“As of May 8, 2001, 21 patients, most with oral lesions, who had been exposed to FMDV during the present outbreak had been investigated by application of PCR to swabs from suspected lesions, and all tested negative. In three of the individuals a human enterovirus was detected that is consistent with a diagnosis of the common benign infection affecting human beings, mainly children—hand, foot, and mouth disease.”	^23^	
	Postulated reasons for human FMD		“The frequency of FMD in man appears to be low and it is evident that predisposing factors must play a crucial part in the initiation of clinical signs. These factors remain largely undetermined but, among other possibilities, crowding and debility, wounds, and a high exposure intensity must be considered important; the reports of many authors indicate that children develop clinical infection more readily than adults. In addition to minor wounds, pre-existing skin conditions, such as dermatitis, keratomata, or tinea, appear to facilitate the establishment of the virus.”	^35^	
Laboratory release	Institute of Animal Health, Pirbright Site, United Kingdom, 2007, 8 infected		“The Pirbright site, comprising the laboratories of the IAH and Merial Animal Health Limited (Merial), is situated 4.4 km from the first IP. Both laboratories were working with the O1 BFS 1860 virus strain, making this site a likely source of the outbreak.”	^65^	No
	FMD outbreak in Germany		“Considering all circumstances of the recent outbreaks, it seems unrealistic to believe the primary infection was not due to the escape of virus from the neighboring vaccine plant. The annual vaccination campaigns since 1970 against FMD were useless because most of the primary outbreaks of FMD since then can be traced to the production or the application of vaccines.”	^66^	
Chemical inactivation	Alcohol-based disinfection (human disinfection)	Product: Vir Stera —Ethanol (76.9–81.4% w/w) phosphoric acid (0.6% w/w)—pH 2.8, dilution 1 × Contact time: 0.5 min Product: Alpet NV—Ethanol (50.0% w/w)—Citric acid (0.5% w/w)—pH 4.1, dilution 1 × Contact time: 0.5 min	“Vir Stera and Alpet NV, acidic ethanol disinfectants, significantly reduced the infectivity of FMDV by at least 3.75 log10 within 0.5 min of exposure time and had a great effect. FMDV is sensitive to low pH, so that in this case, FMDV was inactivated by the main effect of acidic conditions. On the other hand, Hibiscohol S and Hibiscohol S Gel 1, which are neutral and contain higher concentrations of ethanol and chlorhexidine gluconate, reduced the titer by 2.5–2.75 log10 after 5 min exposure. Hibiscohol SHL, which is neutral and contains lower concentration of ethanol and chlorhexidine gluconate, reduced the titer by 1.5 log10 after 5 min exposure.” “It is presumed that the acidic ethanol disinfectants are effective for human use”	^67^	No
	Alkaline cleaner (environment disinfection)	Product: Degreaser FII—Alkaline agent (4.4% w/w)—pH 12.2, dilution 20 × Contact time: 0.5 min Product: Start clean—Alkaline agent (4.9% w/w)—pH 12.1, dilution 50 × Contact time: 0.5 min	“Two alkaline cleaners, Degreaser FII and Start Clean, significantly reduced the infectivity of FMDV by at least 3 log10 within 0.5 min of exposure time and had a great effect” “Alkaline cleaners are effective for use in the infected environment for the control of a FMD outbreak.”	^67^	
	Citric acid	3% citric acid Contact time: 15 min—nonporous 30 min—porous	“Hard, nonporous, and porous food and non-food surfaces including, but not limited to, the following: any Federal, State, or private indoor or outdoor use site, such as: agricultural and non-agricultural equipment and facilities; transportation equipment and facilities; quarantine equipment and facilities; laboratory equipment and facilities; and footwear/personal protective equipment associated with the above use sites.”	^40^	
	Inactivation by heat and guanidine	5.2 mM guanidine hydrochloride 54°C for 1 h	“A study of the ability of 49 strains of FMD virus to replicate in BHK-21 monolayer cells maintained under a standard agar overlay containing 5.2 mM guanidine hydrochloride and to withstand heat inactivation at 54 degrees C for 1 h showed that strains belonging to serotypes C, O, and Asia 1 were generally more resistant to guanidine and heat stable than the SAT 1, 2, and 3 serotypes. The type A viruses as a whole occupied an intermediate position between these two groups. In vitro passage in BHK-21 cells influenced the guanidine sensitivity of 3 (O, C, and SAT 3) of the 7 FMD serotypes, suggesting that this is not a stable genetic marker. Heat stability of the FMD viruses, however, did not change on passage, suggesting that this is a stable characteristic inherent in any homogeneous FMD virus population.”	^42^	
	Hydrogen peroxide	35% vaporized hydrogen peroxide, Steris	“Validation results for two different hydrogen peroxide fumigation methods and compare these to formaldehyde fumigation. The results identify hydrogen peroxide as a suitable alternative to formaldehyde fumigation.”	^50^	
	Hydrogen peroxide vapor	35% HPV 115 min 3 replicate cycles	“Forty-five biological indicators produced using FMDV cultures of ≥10^6^ TCID_50_ ml^−1^ (Tissue Culture Infective Dose) produced no cytopathic effect (CPE) when introduced to monolayers of BHK21 Cl13 cells after exposure to 35% HPV for a period of 115 minutes in three replicate cycles (15 BIs per cycle to give a total of 45 BIs) conducted over three separate days … The results of this preliminary experimentation suggest that 35% HPV is able to achieve full inactivation of FMDV on a hard surface within a room on a repeatable basis.”	^48,49^	
			“We tested varying dilutions and contact times of aerosolised, hydrogen peroxide (AHP) against FMDV, SVDV and SVA by the standard US EPA and modified methods. AHP was effective against all three viruses, albeit at a higher concentration and double the manufacturer recommended contact time when testing wet films of SVDV.”	^48^	
	Chlorine disinfectant (environment disinfection)	Sodium hypochlorite (6% w/w)—pH 9.5, dilution 300 × Time 0.5 min	“Zia knock, a sodium hypochlorite product (200 ppm), significantly reduced the infectivity of FMDV by at least 3.5 log10 within 0.5 min of exposure time … In this study, we evaluated sodium hypochlorine and it had a great effect against FMDV”	^67^	
	Sodium hypochlorite	3% (3000 ppm) 15 min—nonporous 30 min—porous	“Any porous and nonporous surfaces at any Federal, State, or private indoor or outdoor use sites, including but not limited to, agricultural equipment and facilities; transportation equipment and facilities; quarantine equipment and facilities; laboratory equipment and facilities; and footwear/personal protective equipment associated with the above use sites.”	^40^	
	Sodium hypochlorite and citric acid	1000 ppm sodium hypochlorite and 1% citric acid	“Foot and Mouth Disease virus (FMDV), African Swine Fever virus (ASFV), and Classical Swine Fever virus (CSFV) dried on steel and plastic surfaces.” “ASFV and FMDV were susceptible to sodium hypochlorite (500 and 1000 ppm, respectively) and citric acid (1%) resulting in complete disinfection”	^39^	
	Lonza DC 101 (environment disinfection)	Alkyl dimethyl benzyl ammonium chloride, didecyl dimethyl ammonium chloride, octyl decyl dimethyl ammonium chloride, dioctyl dimethyl ammonium chloride—4 oz/gal (30 g/L)	Lonza DC 101 “Livestock premises, livestock feeding and watering equipment, and livestock equipment” Concentration (product/water) 4 oz/gal; Contact time 30 min.	^40^	
	Maquat MQ615-AS (environment disinfection)	Alkyl dimethyl benzyl ammonium chloride, didecyl dimethyl ammonium chloride, octyl decyl dimethyl ammonium chloride, dioctyl dimethyl ammonium chloride—1 oz/gal (7.5 g/L)	Maquat MQ615-AS “Livestock premises, livestock feeding and watering equipment, livestock equipment, livestock transportation vehicles, hog farrowing houses, hog barns/houses/parlors/pens, farrowing equipment, animal feeding and watering equipment, animal equipment, animal transportation vehicles, and shoe baths” Concentration (product/water) 1 oz/gal; Contact time 10 min.	^40^	
	1% Virkon™ (environment disinfection)	5-min contact time	“A FMDV A24 titre of >5 log_10_ TCID_50_ ml^−1^ was recovered from control stainless steel and pH-adjusted concrete coupons (Table 4). Disinfection with 1% Virkon™ S on stainless steel resulted in a complete kill on all eight coupons (two test days). Disinfection on pH-adjusted Concrete C with 1, 2 and 5% Virkon S also resulted in complete virus inactivation based on the absence of detectable CPE in all replicates.”	^68^	
	Virkon S (environment disinfection)	1.3 oz (1 sachet)/gal	“Animal feed equipment, livestock barns, livestock pens, livestock stalls, livestock stables, livestock equipment, cattle feedlot, hog farrowing pen premises, hog barns/houses/parlors/pens, animal quarters, animal feeding and watering equipment, animal equipment, agricultural premises, agricultural equipment, animal transportation vehicles, and human footwear.” Concentration (product/water) 1.3 oz (1 sachet)/gal; Contact time 10 min.	^40^	
	Formaldehyde (gaseous)	10 g/m^3^ at 70% RH for 10 min OR 3 g/m^3^ for 24 h	“After surface disinfection, fumigation with formaldehyde (10 g/m^3^ at 70% RH) for at least 10 minutes or (3 g/m^3^ for 24 hours or equivalent with other aldehydes, e.g., glutaraldehyde, or ethylene oxide (0.8 g/litre at 50°C for 1.5 hours). Equipment, for example contractors' tool boxes, laptops, etc. which is fumigated out of a Restricted Zone should be cleaned and be opened as much as reasonably possible to allow penetration of the gaseous fumigant”	^13^	
	Formaldehyde (liquid)	4%	“4% formaldehyde or equivalent with other aldehydes, e.g., glutaraldehyde”; The efficiency is considerably improved by the addition of a non-ionic detergent.	^13^	
	Ethylene oxide (gaseous)	0.8 g/L at 50°C for 1.5 h	“After surface disinfection, fumigation with formaldehyde (10 g/m^3^ at 70% RH) for at least 10 minutes or (3 g/m^3^ for 24 hours or equivalent with other aldehydes, e.g., glutaraldehyde, or ethylene oxide (0.8 g/litre at 50°C for 1.5 hours). Equipment, for example contractors' tool boxes, laptops, etc. which is fumigated out of a Restricted Zone should be cleaned and be opened as much as reasonably possible to allow penetration of the gaseous fumigant”	^13^	
Thermal	Autoclave	115°C for 30 min	“Either by steam sterilization within an autoclave, at 115°C for 30 minutes, or an equivalent heat effect”	^13^	No

IU, infectious units; LAIs, laboratory-acquired infections; OPF, oropharyngeal fluid; RH, relative humidity.

### Disinfection and Decontamination

#### Chemical

FMDV is sensitive to acid and alkaline pH conditions. NaOH or sodium carbonate (Na_2_CO_3_) or other alkaline treatment at pH 12 for at least 10 h is sufficient to inactivate FMDV.^13^ Recommended chemical disinfectants include 4% sodium carbonate or 10% washing soda (Na_2_CO_3_ dehydrate), 0.5% caustic soda (NaOH), 0.2% citric acid, 4% formaldehyde, or equivalent with other aldehydes, for example, glutaraldehyde.^13^ Krug et al. demonstrated FMDV dried on steel and plastic surfaces and exposed to 1000 ppm sodium hypochlorite and 1% citric acid was completely inactivated.^39^ The U.S. Department of Agriculture recognizes products containing a mixture of alkyl dimethyl benzyl ammonium chloride, didecyl dimethylammonium chloride, octyl decyl dimethyl ammonium chloride, and dioctyl dimethyl ammonium chloride (i.e., Lonza 101 and Maquat MQ615-AS), and those containing sodium chloride and potassium peroxymonosulfate (i.e., Virkon™ S) as being effective disinfectants that can be used in farm settings.^40^

#### Thermal and autoclaving

FMDV is sensitive to heat. Numerous studies have examined the thermal inactivation of FMDV.^41–45^ Exposure of materials to 100°C for 1 h or an equivalent heat effect is sufficient to inactivate FMDV in the effluent so that no residual infectivity can be detected.^13^ Sterilization by steam using an autoclave at least 115°C for 30 min is also effective for solid and liquid waste, although the system should be validated before use.^13^

#### Gaseous decontamination

Animal facilities and laboratories where FMDV work is performed are usually fumigated by gaseous decontamination before maintenance or decommissioning. Formaldehyde has been the method of choice for room and equipment fumigation for several decades^46,47^ to decontaminate FMDV animal facilities.^13^ Concentrations recommended for effective gaseous decontamination are as follows: formaldehyde at 10 g/m^3^ at 70% relative humidity for at least 10 min or 3 g/m^3^ for 24 h or equivalent with other aldehydes (e.g., glutaraldehyde) or ethylene oxide for 0.8 g/L at 50°C for 1.5 h.^13^ Due to its carcinogenicity, formaldehyde is increasingly replaced by alternative systems, particularly those based on vaporized hydrogen peroxide, an effective disinfectant against FMDV.^48–50^

Evidence regarding the route of inoculation/modes of transmission, infectious dose, laboratory-acquired infections, and disinfection and decontamination strategies is provided in Table 1.

## Knowledge Gaps

### Engineering Controls

Each facility handling FMDV is unique regarding engineering systems (design of ventilation, directional airflow, air exchange rate, humidity, etc.). Since the various experimental test parameters were precise in the studies mentioned above that described FMDV transmission or containment (e.g., exposure time, animal, proximity to animals, activity), the results are difficult to compare. There is little information about the contribution of specific technical measures to safety (e.g., directional airflow in a laboratory vs. animal room, air exchange rate). Is it a combination of all measures or are specific individual measures more critical than others? What minimum technical standards are required for laboratories or animal facilities to operate in a country where FMD is exotic? Which system works in which environments? How many safety layers are needed to mitigate the risks associated with an FMDV activity?

### Requirement for a Shower

Many laboratories that work with FMDV mandate showering out of the facility. It has been demonstrated that showering out of an animal unit where FMDV-infected animals are kept prevents transmission to the outside environment.^21^ The same result was achieved if a change of clothing was combined with additional hygiene measures (hand hygiene, etc.).^21,33^ It should be highlighted that the scientific evidence is available only for in vivo animal work, and no scientific data exist that support showering out after in vitro laboratory work. No evidence demonstrates the requirement for a shower when leaving an FMDV vaccine production facility under normal conditions. However, in the case of large-scale spills and subsequent worker contamination of boots and clothing, it would be expected that decontamination, including personnel showering, is warranted; however, the evidence for the showering parameters (i.e., duration and use of soap/chemicals) needs to be defined.

### Human Nasal Transmission Route Under Experimental Conditions

While not strictly a knowledge gap, only one instance of human nasal transmission of FMDV from infected humans to noninfected animals was recorded by Sellers et al.^27^ under experimental conditions. It is worth noting that this was an artificial infection where FMDV-exposed staff sneezed and coughed at the nostrils of animals for 30 s to induce infection, significantly decreasing the possibility of occurring under natural conditions.^17,51^ There remains no evidence of infection of susceptible animals by humans following in vitro laboratory work with FMDV.

### Use of Respirators or Masks to Prevent Human Transmission

There has been a lively debate on the use of face masks in field conditions.^51–55^ The evidence is inconclusive regarding the effect of respiratory PPE (i.e., surgical mask, FFP2, FFP3, N95) on virus uptake by humans after handling FMDV-infected animals. Further work on the effect of nose blowing and washing nostrils to prevent inadvertent transmission is also required.

### Organizational Measures

The EU-FMD guidelines and many facilities have implemented a quarantine period of 3–5 days after working with FMDV in the laboratory; however, there is no specification on whether this should be applied to both in vivo and in vitro work. The 3-day quarantine rule is based on studies with FMDV-infected large animals during which personnel exposure is most significant and using primary containment is practically impossible.^26^

### Risk Assessment

Many FMDV (and other livestock diseases) facilities are still being built based on the specifications of U.S., Australian, Canadian, or EU guidelines where the FMD is exotic and hence a heightened threat to the domestic livestock industries. Such facilities are costly to construct, provide services such as water and electricity, and maintain general backup and redundancies in case of service failures. This issue is exacerbated as the facility ages and requires increased maintenance and repairs.

**Figure 1.**
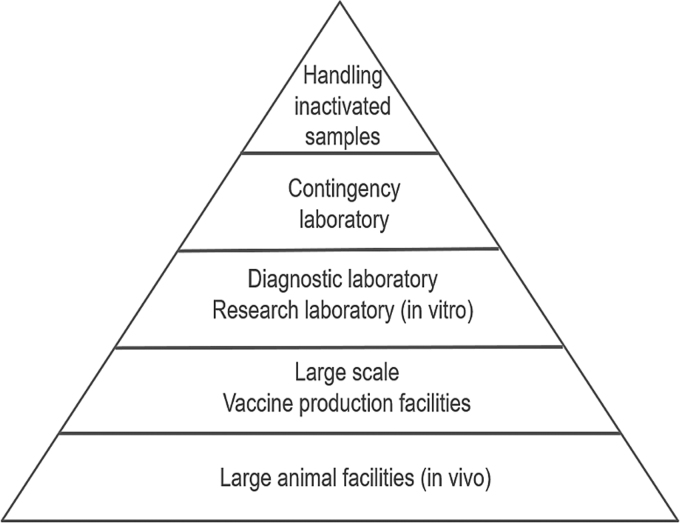
The risk pyramid for the different types of facilities where FMD activities are performed. FMD, foot and mouth disease.

Each facility should establish a risk assessment framework to determine the risk mitigation measures. These measures should be proportionate to the risks encountered in a facility and reflect the local, regional, or national situation. The risk pyramid (Figure 1) demonstrates the different types of facilities where FMDV activities are performed, highlighting that large-scale and large animal facilities involve the most significant risks, and those performing in vitro or diagnostic contingency are of lower risk. Further guidance on risk assessment is required for different FMDV facilities that consider local/regional/national circumstances. Advantages, disadvantages, challenges, and pitfalls of the various mitigation measures on the technical, organizational, and PPE level should be evaluated against each other at the planning stages of a new laboratory facility.

### Disinfection and Decontamination

#### Fumigation

Animal facilities and laboratories where FMDV work is ongoing are usually fumigated before maintenance or decommissioning. Formaldehyde has been the method of choice for room and equipment fumigation for decades^46^ due to its effectiveness and relative simplicity. Still, due to concerns regarding the carcinogenic nature of formaldehyde, relevant publications have demonstrated that fumigation with alternative chemicals, such as vaporized hydrogen peroxide, is an effective disinfectant against FMDV.^48,49^ Nevertheless, further studies comparing the different strains of FMDV are still required. In addition, there are still many gaps in how facilities, including all engineering systems, are decontaminated and/or decommissioned to be safe for further use.

## Conclusions

Due to the highly contagious nature of FMDV and the severe economic consequences of incursions into FMD-free regions, most countries opt for highly engineered biocontainment facilities when performing in vitro or in vivo FMDV laboratory activities. Taking a “belt and suspenders” approach may appear prudent; however, without thoroughly examining the actual risks involved with laboratory activities can result in an overly complex system that is technically complex and financially unsustainable. However, handling FMDV in an endemic country may not represent the same risk profile as in a country where the disease is exotic and depends on the nature of the activities performed (i.e., PCR diagnostics, *cf.* in vitro virus isolation). A thorough risk assessment, including factors such as the local/national situation, could help design facilities that fit the purpose in a specific region or country.

## References

[1] Blacksell SD, Dhawan S, Kusumoto M, et al. The Biosafety Research Road Map: The search for evidence to support practices in human and veterinary laboratories. Appl Biosaf 2023;28(2):1–8. 37342514 10.1089/apb.2022.0040 PMC10277988

[2] Donaldson AI. The influence of relative humidity on the stability of foot-and-mouth disease virus in aerosols from milk and faecal slurry. Res Vet Sci 1973;15(1):96–101. 4360318

[3] Donaldson AI. FMD control strategies. Vet Rec 2001;148(22):700. 11425260

[4] Donaldson AI, Gloster J, Harvey LD, et al. Use of prediction models to forecast and analyse airborne spread during the foot-and-mouth disease outbreaks in Brittany, Jersey and the Isle of Wight in 1981. Vet Rec 1982;110(3):53–57; doi: 10.1136/vr.110.3.53 7064324

[5] Paton DJ, Gubbins S, King DP. Understanding the transmission of foot-and-mouth disease virus at different scales. Curr Opin Virol 2018;28:85–91; doi: 10.1016/j.coviro.2017.11.013 29245054

[6] Blacksell SD, Siengsanan-Lamont J, Kamolsiripichaiporn S, et al. A history of FMD research and control programmes in Southeast Asia: Lessons from the past informing the future. Epidemiol Infect 2019;147:e171; doi: 10.1017/S0950268819000578 31063108 PMC6499730

[7] Rweyemamu M, Roeder P, Mackay D, et al. Epidemiological patterns of foot-and-mouth disease worldwide. Transbound Emerg Dis 2008;55(1):57–72; doi: 10.1111/j.1865-1682.2007.01013.x 18397509

[8] European Commission for the Control of Foot and Mouth Disease (Eu-FMD). The Progressive Control Pathway for Foot and Mouth Disease (PCP-FMD); 2021. Available from: www.fao.org/eufmd/global-situation/pcp-fmd/en/ [Last accessed: April 13, 2023].

[9] European Union Health and Food Safety Directorate-General. Bio-Risk Management in Laboratories Handling Live FMD Virus. EFSA: Luxembourg; 2015.

[10] European Commission for the Control of Foot and Mouth Disease (Eu-FMD). Minimum biorisk management standards for laboratories working with foot and mouth disease; 2013. Available from: www.fao.org/fileadmin/user_upload/eufmd/Lab_guidelines/FMD_Minimumstandards_2013_Final_version.pdf [Last accessed: April 13, 2023].

[11] Centers for Disease Control and Prevention. Biosafety in Microbiological and Biomedical Laboratories (BMBL) 6th Edition. Centers for Disease Control and Prevention: Atlanta, GA; 2020.

[12] Federal Select Agent Program. Select Agents and Toxins List; 2021. Available from: https://www.selectagents.gov/sat/list.htm [Last accessed: April 13, 2023].

[13] European Commission for the Control of Foot and Mouth Disease (Eu-FMD). 38th General Session of the EuFMD Commission, April 28–30, 2009. Rome; 2009. Available from: https://www.fao.org/3/bs321e/bs321e.pdf [Last accessed: April 13, 2023].

[14] United States Department of Agriculture Animal and Plant Health Inspection Service. Foot-and-Mouth Disease; 2021. Available from: https://www.aphis.usda.gov/publications/animal_health/fs-fmd-general.pdf [Last accessed April 13, 2023].

[15] Wong CL, Yong CY, Ong HK, et al. Advances in the diagnosis of foot-and-mouth disease. Front Vet Sci 2020;7:477; doi: 10.3389/fvets.2020.00477 32974392 PMC7473413

[16] Alexandersen S, Mowat G. Foot-and-Mouth Disease: Host Range and Pathogenesis. In: Foot-and-Mouth Disease Virus. (Mahy B. ed.) Springer-Verlag: Heidelberg; 2005; pp. 9–42. 10.1007/3-540-27109-0_2 15648173

[17] Auty H, Mellor D, Gunn G, et al. The risk of foot and mouth disease transmission posed by public access to the countryside during an outbreak. Front Vet Sci 2019;6:381; doi: 10.3389/fvets.2019.00381 31750321 PMC6848457

[18] Botner A, Belsham GJ. Virus survival in slurry: Analysis of the stability of foot-and-mouth disease, classical swine fever, bovine viral diarrhoea and swine influenza viruses. Vet Microbiol 2012;157(1–2):41–49; doi: 10.1016/j.vetmic.2011.12.010 22226541

[19] Donaldson AI. The influence of relative humidity on the aerosol stability of different strains of foot-and-mouth disease virus suspended in saliva. J Gen Virol 1972;15(1):25–33; doi: 10.1099/0022-1317-15-1-25 4337217

[20] Donaldson AI, Ferris NP. The survival of foot-and-mouth disease virus in open air conditions. J Hyg (Lond) 1975;74(3):409–416; doi: 10.1017/s002217240004691x 168250 PMC2130593

[21] Amass SF, Mason PW, Pacheco JM, et al. Procedures for preventing transmission of foot-and-mouth disease virus (O/TAW/97) by people. Vet Microbiol 2004;103(3–4):143–149; doi: 10.1016/j.vetmic.2004.07.020 15504585

[22] Armstrong R, Davie J, Hedger RS. Foot-and-mouth disease in man. Br Med J 1967;4(5578):529–530; doi: 10.1136/bmj.4.5578.529 4294412 PMC1749100

[23] David W, Brown G. Foot and mouth disease in human beings. Lancet 2001;357(9267):1463; doi: 10.1016/s0140-6736(00)04670-5 11377592

[24] Dlugosz H. Foot-and-mouth disease in man. Br Med J 1943;1(4284):189–190; doi: 10.1136/bmj.1.4284.189 20784684 PMC2282216

[25] Donaldson A, Knowles N. Foot-and-mouth disease in man. Vet Rec 2001;148(10):319. 11315144

[26] Sellers RF, Donaldson AI, Herniman KA. Inhalation, persistence and dispersal of foot-and-mouth disease virus by man. J Hyg (Lond) 1970;68(4):565–573; doi: 10.1017/s0022172400042492 4321595 PMC2130876

[27] Sellers RF, Herniman KA, Mann JA. Transfer of foot-and-mouth disease virus in the nose of man from infected to non-infected animals. Vet Rec 1971;89(16):447–449; doi: 10.1136/vr.89.16.447-a 4332392

[28] Wright CF, Gloster J, Mazelet L, et al. Short-lived carriage of foot-and-mouth disease virus in human nasal cavities after exposure to infected animals. Vet Rec 2010;167(24):928–931; doi: 10.1136/vr.c6275 21262692

[29] Donaldson AI, Alexandersen S. Predicting the spread of foot and mouth disease by airborne virus. Rev Sci Tech 2002;21(3):569–575; doi: 10.20506/rst.21.3.1362 12523697

[30] Donaldson AI, Ferris NP. Sites of release of airborne foot-and-mouth disease virus from infected pigs. Res Vet Sci 1980;29(3):315–319. 6265993

[31] Donaldson AI, Gibson CF, Oliver R, et al. Infection of cattle by airborne foot-and-mouth disease virus: Minimal doses with O1 and SAT 2 strains. Res Vet Sci 1987;43(3):339–346. 2832913

[32] Donaldson AI, Herniman KA, Parker J, et al. Further investigations on the airborne excretion of foot-and-mouth disease virus. J Hyg (Lond) 1970;68(4):557–564; doi: 10.1017/s0022172400042480 4321594 PMC2130857

[33] Amass SF, Pacheco JM, Mason PW, et al. Procedures for preventing the transmission of foot-and-mouth disease virus to pigs and sheep by personnel in contact with infected pigs. Vet Rec 2003;153(5):137–140; doi: 10.1136/vr.153.5.137 12934795

[34] Bauer K. Foot-and-Mouth Disease as Zoonosis. In: Viral Zoonoses and Food of Animal Origin. (Kaaden OR, Czerny CP, Eichhorn W. eds.) Springer-Verlag: Vienna; 1997; pp. 95–97. 10.1007/978-3-7091-6534-8_9 9413529

[35] Hyslop NSG. Transmission of the virus of foot and mouth disease between animals and man. Bull World Health Organ 1973;49(6):577–585. 4374322 PMC2481018

[36] Pape J. A contribution to human foot-and-mouth disease [in German]. Ber Tier Woch 1921;33:354.

[37] Prempeh H, Smith R, Muller B. Foot and mouth disease: The human consequences. The health consequences are slight, the economic ones huge. BMJ 2001;322(7286):565–566; doi: 10.1136/bmj.322.7286.565 11238137 PMC1119772

[38] Wisniewski J, Jankowska J. Asymptomatic infection with foot-and-mouth disease in humans [in Polish]. Przegl Epidemiol 1969;23(2):263–267. 4308462

[39] Krug PW, Lee LJ, Eslami AC, et al. Chemical disinfection of high-consequence transboundary animal disease viruses on nonporous surfaces. Biologicals 2011;39(4):231–235; doi: 10.1016/j.biologicals.2011.06.016 21798759

[40] United States Department of Agriculture Animal and Plant Health Inspection Service. Disinfectants Approved for Use Against Foot-and-Mouth Disease Virus in Farm Settings; 2020. Available from: https://www.aphis.usda.gov/animal_health/emergency_management/downloads/fmd-virus-disinfectants.pdf [Last accessed: April 13, 2023].

[41] Pagliaro AF, Masana MO, Sanjurjo ED, et al. Foot-and-mouth disease virus inactivation in miniburgers by a continuous dry-moist heat cooking system. J Food Prot 1996;59(2):181–184; doi: 10.4315/0362-028X-59.2.181 31159010

[42] Nettleton PF, Davies MJ, Rweyemamu MM. Guanidine and heat sensitivity of foot-and-mouth disease virus (FMDV) strains. J Hyg (Lond) 1982;89(1):129–138; doi: 10.1017/s0022172400070625 6284836 PMC2134169

[43] Kamolsiripichaiporn S, Subharat S, Udon R, et al. Thermal inactivation of foot-and-mouth disease viruses in suspension. Appl Environ Microbiol 2007;73(22):7177–7184; doi: 10.1128/AEM.00629-07 17660312 PMC2168231

[44] Gubbins S, Forster J, Clive S, et al. Thermal inactivation of foot and mouth disease virus in extruded pet food. Rev Sci Tech 2016;35(3):965–972; doi: 10.20506/rst.35.3.2582 28332656

[45] de Leeuw PW, Tiessink JW, van Bekkum JG. Aspects of heat inactivation of foot-and-mouth disease virus in milk from intramammarily infected susceptible cows. J Hyg (Lond) 1980;84(2):159–172; doi: 10.1017/s0022172400026668 6244342 PMC2133889

[46] Dreyfus W. Review of formaldehyde fumigation. Am J Pub Health 1914;4(11):1046–1049. 10.2105/ajph.4.11.1046 PMC1286481 18009132

[47] Kümin D, Albert MG, Summermatter K. Case study: Room fumigation using aerosolized hydrogen peroxide—A versatile and economic fumigation method. Appl Biosaf 2019;24(4):200–206; doi: 10.1177/1535676019887049 36032058 PMC9134476

[48] Hole K, Ahmadpour F, Krishnan J, et al. Efficacy of accelerated hydrogen peroxide((R)) disinfectant on foot-and-mouth disease virus, swine vesicular disease virus and Senecavirus A. J Appl Microbiol 2017;122(3):634–639; doi: 10.1111/jam.13361 27886439

[49] Petit BM, Almeida FC, Uchiyama TR, et al. Evaluating the efficacy of hydrogen peroxide vapour against foot-and-mouth disease virus within a BSL-4 biosafety facility. Lett Appl Microbiol 2017;65(4):281–284. 28736948 10.1111/lam.12778

[50] Kümin D, Albert MG, Summermatter K. Comparison and validation of three fumigation methods to inactivate foot-and-mouth disease virus. Appl Biosaf 2018;23(2):70–76; doi: 10.1177/1535676018771982

[51] Donaldson AI. Foot-and-mouth disease and the use of face masks. Vet Rec 2008;162(5):163; doi: 10.1136/vr.162.5.163 18245752

[52] Donaldson AI. Foot-and-mouth disease and the use of face masks. Vet Rec 2008;162(2):62; doi: 10.1136/vr.162.2.62-a 18192662

[53] Jones TO. Foot-and-mouth disease and the use of face masks. Vet Rec 2007;161(25):863–864. 18156598

[54] Jones TO. Foot-and-mouth disease and the use of face masks. Vet Rec 2008;162(4):131–132; doi: 10.1136/vr.162.4.131-a 18223274

[55] Sellers B. Foot-and-mouth disease and the use of face masks. Vet Rec 2008;162(7):224; doi: 10.1136/vr.162.7.224 18281637

[56] Bravo de Rueda C, de Jong MC, Eble PL, et al. Quantification of transmission of foot-and-mouth disease virus caused by an environment contaminated with secretions and excretions from infected calves. Vet Res 2015;46:43; doi: 10.1186/s13567-015-0156-5 25928658 PMC4404111

[57] Bartley LM, Donnelly CA, Anderson RM. Review of foot-and-mouth disease virus survival in animal excretions and on fomites. Vet Rec 2002;151(22):667–669; doi: 10.1136/vr.151.22.667 12498410

[58] Fukai K, Nishi T, Morioka K, et al. Horizontal transmission of foot-and-mouth disease virus O/JPN/2010 among different animal species by direct contact. Transbound Emerg Dis 2020;67(1):223–233; doi: 10.1111/tbed.13344 31482692

[59] Pacheco JM, Arzt J, Rodriguez LL. Early events in the pathogenesis of foot-and-mouth disease in cattle after controlled aerosol exposure. Vet J 2010;183(1):46–53; doi: 10.1016/j.tvjl.2008.08.023 18930417

[60] Donaldson A. Airborne spread of foot-and-mouth disease. Microbiol Today 1999;26:118–119.

[61] Donaldson AI, Ferris NP, Gloster J. Air sampling of pigs infected with foot-and-mouth disease virus: comparison of Litton and cyclone samplers. Res Vet Sci 1982;33(3):384–385. 6296955

[62] Moreno-Torres KI, Brito BP, Branan MA, et al. Foot-and-mouth disease infection dynamics in contact-exposed pigs are determined by the estimated exposure dose. Front Vet Sci 2018;5:167; doi: 10.3389/fvets.2018.00167 30079340 PMC6062637

[63] Sellers R, Gloster J. Foot-and-mouth disease: A review of intranasal infection of cattle, sheep and pigs. Vet J 2008;177(2):159–168; doi: 10.1016/j.tvjl.2007.03.009 17509917

[64] Alexandersen S, Donaldson AI. Further studies to quantify the dose of natural aerosols of foot-and-mouth disease virus for pigs. Epidemiol Infect 2002;128(2):313–323; doi: 10.1017/s0950268801006501 12002550 PMC2869825

[65] Cottam EM, Wadsworth J, Shaw AE, et al. Transmission pathways of foot-and-mouth disease virus in the United Kingdom in 2007. PLoS Pathog 2008;4(4):e1000050; doi: 10.1371/journal.ppat.1000050 18421380 PMC2277462

[66] Strohmaier K. Conclusions from the outbreak of foot-and-mouth disease in the government district of Hannover in 1987/1988 [in German]. Dtsch Tierarztl Wochenschr 1990;97(5):210–212. 2194777

[67] Harada Y, Lekcharoensuk P, Furuta T, et al. Inactivation of foot-and-mouth disease virus by commercially available disinfectants and cleaners. Biocontrol Sci 2015;20(3):205–208; doi: 10.4265/bio.20.205 26412701

[68] Gabbert LR, Neilan JG, Rasmussen M. Recovery and chemical disinfection of foot-and-mouth disease and African swine fever viruses from porous concrete surfaces. J Appl Microbiol 2020;129(5):1092–1101; doi: 10.1111/jam.14694 32379950 PMC7687137

